# Molecular Cloning and Functional Analysis of *IrUGT86A1-like* Gene in Medicinal Plant *Isodon rubescens* (Hemsl.) Hara

**DOI:** 10.3390/life12091334

**Published:** 2022-08-28

**Authors:** Conglong Lian, Jinxu Lan, Bao Zhang, Hao Yang, Kaihua Guo, Jingjing Li, Suiqing Chen

**Affiliations:** School of Pharmacy, Henan Key Laboratory of Chinese Medicine Resources and Chinese Medicine Chemistry, Henan University of Chinese Medicine, Boxue Road, Jinshui District, Zhengzhou 450046, China

**Keywords:** *Isodon rubescens*, UDP-glycosyltransferase, RT-qPCR, prokaryotic expression

## Abstract

The synthesis of secondary metabolites in plants often includes glycosylation modifications. Often, the final step of constructing plant secondary metabolites is completed by glycosylation transferases, which are also involved in many cell processes. In this study, a UDP-glycosyltransferase gene (*UGT*) was amplified from *Isodon rubescens* (Hemsl.) Hara with RT-PCR and named *IrUGT86A1-like* (GenBank: MZ913258). Here, we found that *IrUGT86A1-like* gene is 1450 bp in length and encodes for 479 amino acids. Bioinformatics analysis revealed that *IrUGT86A1-like* is a stable and hydrophilic protein, located in the cytoplasm with a transmembrane domain. Phylogenetic analysis showed that IrUGT86A1-like protein has the closest genetic relationship with the UDP-glycosyltransferase 86A1-like protein (XP_042054241.1) of *Salvia splendens*. RT-qPCR analysis demonstrated that the expression of *IrUGT86A1-like* gene varied in different tissues; leaves had the highest expression followed by flowers, stems, and roots had the lowest expression. This expression trend is similar to the distribution of oridonin content in different tissues of *I. rubescens*. Additionally, *IrUGT86A1-like* gene was found to be positively enhanced by NaCl and MeJA treatment, and in contrast was down-regulated by ABA treatment. Finally, the prokaryotic expression vector pEASY^®^-Blunt E1-IrUGT86A1 was successfully used to express about 53 KD of IrUGT86A1-like protein. This research builds a foundation for further investigation on the function of this gene in the synthesis and modification of secondary metabolites.

## 1. Introduction

The “Donglingcao”, a Chinese herb, refers to the dry aboveground part of the Labiatae plant *Isodon rubescens* (Hemsl.) Hara, and is a commonly used for its medicinal properties. Harvested in summer and autumn when the stems and leaves are lush, “Donglingcao” tastes both bitter and sweet, and is consumed slightly cold [1]. It is used for inflammation, antipyretic detoxification, promoting blood circulation, promoting stomach health, and anti-tumor effects. The herb is commonly used in the treatment of sore throat, bronchitis, acute laryngitis, acute suppurative tonsillitis, abdominal mass, and snake bite, [1,2]. Modern pharmacological studies have shown that the main active ingredients in *I. rubescens* plants are diterpene components, oridonin, ponicidin, and water-soluble components, such as rosmarinic acid, among which oridonin is the most active component in the pharmacopoeia [3]. These active ingredients of *I. rubescens* confer significant curative effects on breast cancer, esophageal cancer, colon cancer, and other cancers [4]. Therefore, as an anti-tumor plant with evidence of strong pharmacological activity and low hepatorenal toxicity, *I. rubescens* has been a research focus of pharmacological scientists worldwide regarding the development of molecular pharmacognosy technology and the analysis of the biosynthesis of the main components of *I. rubescens*, including oridonin [5]. In addition to *I. rubescens*’ medicinal properties, it is also a strong and hardy plant with characteristics such as drought resistance, cold resistance, barren resistance, developed root system, and soil amendments and water conservation effects [6].

A form of translational and post-translational modification, glycosylation is an enzyme-mediated process in which sugars are added to proteins or lipids [7]. Glycosylation is not only a key factor in maintaining the stability of the intracellular environment; it also plays an important role in the diversity and complexity of plant secondary metabolites synthesis. Specifically, glycosyltransferases (GTs) are the main enzymes in glycosylation [8,9]. In living organisms, catalyzed activated sugars are connected to different receptor molecules, which contributes to the hypothesis that obtained glycosylation products have many biological functions, and thus regulate various biological functions of plants [9,10]. Among them, GT1 plants are the largest of the GT family. As GT1s use uridine diphosphate-glycosyltransferases as a donor molecule, GT1s are also known as “UGTs” [11]. Previous studies in plants have shown that UGTs are involved in the biosynthesis of terpenoids, phenols, flavonoids, steroids, and other natural products [12]. Furthermore, UGTs catalyze the glycosyltransferase-mediated conversion of uridine diphosphate-activated sugars to substrates such as hormones and secondary metabolites, thus contributing to diverse and complex substrates in plants, which in turn regulate various biological processes such as plant growth, development, disease resistance, and abiotic stress resistance [13,14,15]. In *Arabidopsis*, *UGT79B1* and *UGT91A1* are specifically involved in the modification processing steps of anthocyanin synthesis, and they also contribute to cold, salt, and drought stress tolerance [16]. Similarly, in wheat, overexpression of *Ta-UGT3* gene significantly enhances *Fusarium Head* Blight resistance [17]. Moreover, in rice, a UDP-glucosyltransferase gene regulates rice grain size and biological stress response by changing the reflow of plant metabolic flow [18]. Additionally, *UGT85C2*, *UGT74G1,* and *UGT76G1* in *Stevia rebaudiana*, have been found to have activity in the synthesis of stevioside and rebaudioside A, which are two main glycosides of *Stevia* [19]. Furthermore, a UDP-Glucosyltransferase gene was found to be beneficial to the biosynthesis of flavonoid glycosides in *Cyclocarya paliurus* [20].

Because of the high abundance of terpenoids, flavonoids, alkaloids, steroids, volatile oils, amino acids, organic acids, monosaccharides, and other components in *I. rubescens* [21], glycosylation plays an important role in these plants as it is essential to the synthesis and modification of these compounds. However, research on glycosyltransferase in *I. rubescens* has not yet been reported. Therefore, it is essential to investigate the glycosyltransferase of *I. rubescens* in order to understand the biosynthesis of important secondary metabolites. In a previous transcriptome data analysis with two tissues: callus with undetectably low levels oridonin and adventitious buds with oridonin, a significant difference in the expression in UDP-Glucosyltransferase (*UGT*) gene was found, suggesting that it may modify and regulate oridonin synthesis. With these previous transcriptomic analysis results, this significant differential expression in the *UGT* sequence was used as a reference, and this *UGT* candidate gene was cloned by RT-PCR. The physicochemical properties, protein structure, and evolutionary relationship were analyzed by the bioinformatics. The differential expression in different tissues and responses to different abiotic stresses were further analyzed with fluorescent quantitative PCR (RT-qPCR). Finally, the protein was successfully expressed in *E. coli* bacteria. Overall, this foundational research enables further investigation into the role of the enzyme system encoded by *IrUGT86A1-like* gene in the synthesis of active ingredients in *I. rubescens* and research on the function of this gene with genetic engineering.

## 2. Materials and Methods

### 2.1. Plant Material and Sample Preparation

*I. rubescens* plants were cultivated in artificial intelligence climate boxes with the following conditions: nutrient soil and vermiculite (2:1), photoperiod 14 h/10 h (light/dark), relative humidity 60%, and culture temperature 25 °C. After six months of growth at flowering, the roots, stems, leaves, and flowers of *I. rubescens* were collected for quantification of oridonin and RNA extraction (stored at −80 °C). For the simulated salt stress treatment, uniformly developed seedlings of *I. rubescens* were fully watered using a 200 mM NaCl solution, and the leaves between three and six nodes were harvested at 0, 2, 8, 12, 24, and 72 h. For the MeJA and ABA treatments, 200 µmol MeJA, and 300 µmol ABA, were evenly sprayed on the leaves of *I. rubescens*, respectively, and the leaves between three and six nodes were harvested at six time points at 0, 2, 4, 8, 12, and 24 h. All collected samples were frozen in liquid nitrogen immediately and then preserved at −80 °C for RNA extraction and expression analysis of the *IrUGT86A1-like* gene.

### 2.2. Total RNA Extraction and cDNA Reverse Transcription

Frozen samples of roots, stems, leaves, flowers, and other tissues of *I. rubescens* were individually ground into a fine powder with a pestle and mortar using liquid nitrogen. The total RNA was extracted according to the EASYspin Plus plant RNA extraction kit protocol (AidLab, Beijing, China). An ultramicro spectrophotometer was used to measure quality and quantity of RNA, and all RNA samples were stored at −80 °C refrigerator for downstream analysis. Next, the reverse transcription kit BCS HIScript^TM^ AII-in-One Mix with dsDNAse was to remove genomic DNA contamination, and then to synthesize the first strand of cDNA was synthesized via reverse transcription. The obtained cDNA was adjusted to the corresponding concentration according to quantification with the ultramicro spectrophotometer and stored in a refrigerator at −20 °C for further use.

### 2.3. Cloning of IrUGT86A1-like Gene

First, primers of UGT-F and UGT-R were designed, and the first strand of *I. rubescens* cDNA was used in cloning to obtain *IrUGT86A1-like* gene sequence. The PCR reaction consisted of 2× Taq Plus Master Mix 12.5 μL, cDNA 1 μL, UGT-F 1 μL, UGT-R 1 μL, and ddH_2_O 9.5 μL. The PCR procedure was as follows: pre-denaturation at 95 °C for 3 min; denaturation at 95 °C for 15 s, annealing at 53 °C for 30 s, extension at 72 °C for 90 s, 35 cycles; and extension at 72 °C for 10 min. The PCR products were detected with 1% agarose gel electrophoresis in which DNA was separated based on length, and target DNA band was recovered with SanPrep Column DNA Gel Extraction Kit (Sangon Biotech, Shanghai, China). The obtained target fragment was further ligated using the cloning vector pEASY^®^-Blunt Zero Cloning Kit (Transgen, Beijing, China), and transformed into *E. coli* T1 competent cells. The positive clones were selected and sent to Sangon Biotech (Shanghai) Co., Ltd. for sequencing, and the correct pEASY-Blunt-IrUGT86A1 prokaryotic strain was obtained. The primers for PCR are included in Table S1.

### 2.4. Bioinformatics Analysis

DNAMAN6.0 software was used to analyze sequencing results and investigate and predict proteins. Conserved domain prediction was performed with the NCBI online tool Conserved Domains (https://www.ncbi.nlm.nih.gov/cdd, accessed on 6 May 2022). Protein physical and chemical properties were predicted using the online tool ExPASy (http://www.expasy.ch/tools/protparam.html, accessed on 6 May 2022). Subcellular localization was predicted and analyzed using the online tools WoLF PSORT (https://wolfpsort.hgc.jp/, accessed on 6 May 2022) and PredictProtein (https://predictprotein.org/, accessed on 6 May 2022) results. Protein signal peptides were predicted using online tools SignalP 5.0 Server (http://www.cbs.dtu.dk/services/SignalP/, accessed on 7 May 2022) and TargetP-2.0 (http://www.cbs.dtu.dk/services/TargetP/, accessed on 7 May 2022). The protein transmembrane domain was predicted with TMPred (https://www.expasy.org/resources/tmpred, accessed on 7 May 2022) and the TMHMM Server v.2.0 (http://www.cbs.dtu.dk/services/TMHMM/, accessed on 7 May 2022). Protein secondary structure and coil structure were analyzed using the online tools PredictProtein (https://predictedprotein.org/, accessed on 8 May 2022) and COILS (https://embnet.vital-it.ch/software/COILS_form.html, accessed on 8 May 2022). Three-dimensional modeling of protein domains was performed using SWISS-MODEL (http://swissmodel.expasy.org/, accessed on 8 May 2022).

### 2.5. Multi-Sequence Alignment and Construction of Phylogenetic Tree

Similar amino acid sequences to the *IrUGT86A1-like* gene encoding protein of *I. rubescens* were obtained using blastp on the NCBI database, and the amino acid sequences of UGT86A1 from other plants were downloaded from NCBI database. These UGT86A1 protein sequences including IrUGT86A1-like of *I. rubescens* were processed with to multiple sequence alignment analysis using MEGA7.0 software [22], and the phylogenetic tree was constructed using the neighbor-joining method with the following parameters: bootstrap = 1000 repeat times, and default values were used for other parameters.

### 2.6. Fluorescence Quantitative Expression Analysis

The cDNA of roots, stems, leaves, flowers, and callus of *I. rubescens* were used as templates, while *GADPH* was used as the internal reference gene for RT-qPCR of the *IrUGT86A1-like* gene. The reaction system was as follows: 10 μL of 2 × TransStart^®^ Top Green qPCR SuperMix, 0.4 μL of 50 × Passive Reference Dye II, and 0.4 μL of forward and reverse primers (10 μM), respectively, sample cDNA 2 μL, and ddH_2_O 6.8 μL. Reaction conditions were the following: 94 °C 30 s; 94 °C 5 s, 60 °C 30 s, and 45 cycles. qPCR was repeated three times, and the relative expression of genes was calculated by 2^−ΔΔCt^ method [23]. The primers for RT-qPCR are included in Table S1.

### 2.7. Determination of Oridonin in I. rubescens

Oridonin was extracted and detected according to the reference: Pharmacopoeia of the People’s Republic of China [1]. The roots, stems, leaves, and flowers of *I. rubescens* plants were respectively placed in an oven at 60 °C and baked to a constant weight. Then they were powdered with a mortar. We accurately weighed 1 g of this product powder, put it in a conical flask with a stopper, accurately added 50 mL methanol, weighed and set the weight, placed it for 30 min, gave it ultrasonic treatment (power 250 W, frequency 40 kHz) for 30 min, made up the lost weight with methanol, shook it well, and filtered 0.22 μm to obtain the sample to be tested. Preparation of control solution: an appropriate amount of isodon reference solution was taken, accurately weighed, and methanol was added to make a solution containing 60 μg per 1 mL. An amount of 10 μL of each of the control solution and the test solution were precisely absorbed and injected into the liquid chromatograph for determination. The chromatographic conditions were the following: Waters e2695/2998, Phenomenex C18 column (250 mm × 4.6 mm, 4 um), mobile phase methanol–water (55:45), detection wavelength 239 nm, flow rate 0.8 mL·min^−1^, and column temperature 25 °C.

### 2.8. Prokaryotic Expression Analysis

Using the sequence of the *IrUGT86A1-like* gene and characteristics of the pEASY^®^-Blunt E1 Expression Vector, a pair of prokaryotic expression primers UGT-YF and UGT-YR for *IrUGT86A1-like* gene were designed with DNAMAN software. The ORF sequence of *IrUGT86A1-like* gene was amplified with the TransStart^®^ FastPfu Fly DNA Polymerase High Fidelity DNA Polymerase Kit (TransGen, Beijing, China) and cloned into the prokaryotic expression vector pEASY^®^-Blunt E1 Expression Vector (TransGen, Beijing, China). The recombinant prokaryotic expression vector pEASY-Blunt E1-IrUGT86A1 with 6×HIS-IrUGT86A1-like fusion protein was obtained with PCR identification, sequencing identification, and transformed into BL21 (DE3) prokaryotic expression competent cells. Protein expression was induced by 0.2 mmol/L IPTG for 16 h in the constant temperature vibration incubator with 200 rpm at 18 °C, and SDS-PAGE protein electrophoresis was performed to analyze the expression results.

## 3. Results

### 3.1. Cloning of IrUGT86A1-like Gene from I. rubescens

**Figure 1:**
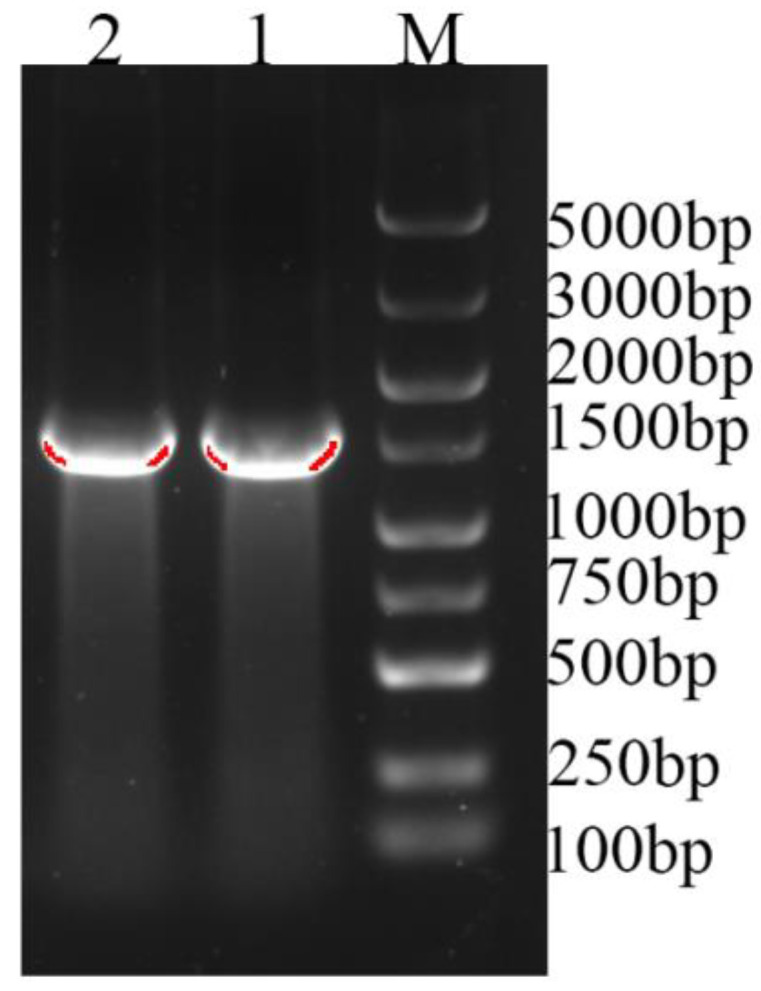
PCR amplification result of *IrUGT86A1-like* gene. M: 5000 ladder marker; 1–2: Amplification bands of *IrUGT86A1-like*.

A *UGT* candidate gene sequence was obtained with bioinformatics analysis of second-generation transcriptome data. Primers were designed and the cDNA from *I. rubescens* leaves was used as the template for cloning. RT-PCR gel electrophoresis results confirmed that the target fragment of about 1500 bp was amplified (Figure 1). Sequencing results showed that the obtained band was 1450 bp in length, containing a 10 bp 5′ untranslated region (5′ UTR) and a 1440 bp open reading frame (ORF), which encodes for 479 amino acids. The blastp comparison results from NCBI showed that the cloned *IrUGT86A1-like* gene had high homology (query coverage greater than 95%) with UDP-glycosyltransferase 86A1-like genes in the database, indicating that the successfully cloned gene is a *UGT* gene of *I. rubescens* and accordingly was named *IrUGT86A1-like*. Sequences are available on NCBI (Genbank Accession Number: MZ913258).

### 3.2. Physicochemical Properties and Localization Analysis of IrUGT86A1-like Protein

ExPASy analysis showed that IrUGT86A1-like protein encodes 479 amino acids, has a molecular weight of 53.03 kD, theoretical isoelectric point of 5.33, molecular formula of C_2404_H_3723_N_633_O_708_S_15_, and contains 7483 atoms. There were 59 negatively charged residues (Asp + Glu) and 41 positively charged residues (Arg + Lys). The instability coefficient is 36.16, the fat solubility coefficient is 94.82, and half-life length in *E. coli* is >10 h. The total average hydrophilicity is −0.057 indicating that the protein encoded by IrUGT86A1-like protein is a stable hydrophilic protein. Signal peptide analysis of SignalP-5.0 and TargetP-2.0 did not detect a signal peptide on this protein. WoLF PSORT protein subcellular localization prediction revealed that there are eight localization sites in the cytoplasm, four sites in the chloroplast, and two sites in the nucleus. Further analysis of protein subcellular localization with PredictProtein predicted that the protein is also located in the cytoplasm (Table S2).

**Figure 2:**
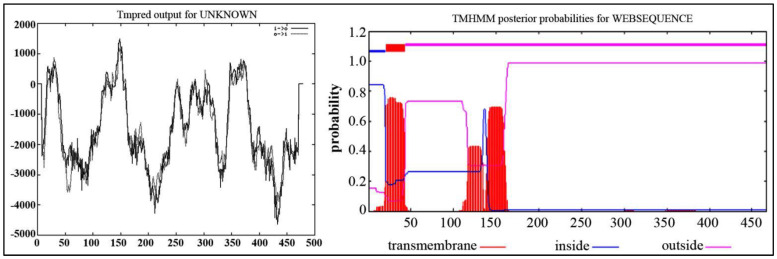
Transmembrane structure prediction of IrUGT86A1-like protein by TMpred (**A**) and TMHMM Server (**B**).

The program TMpred was further used to predict the transmembrane region of IrUGT86A1-like protein. The results showed that there are seven transmembrane regions from inside to outside, and six transmembrane regions from outside to inside. This predicted transmembrane topological model contained four strong transmembrane helix regions at the N-terminal, namely, the inner–outer transmembrane helix region at 23~41 aa, the outer–inner transmembrane helix region at 140~158 aa, the inner–outer transmembrane helix region at 339~364 aa, and the outer–inner transmembrane helix region at 364~387 aa (Figure 2A). The prediction results with the TMHMM Server v. 2.0 revealed that there is a transmembrane region of N-terminal in the cytoplasm (Figure 2B).

### 3.3. Structural Analysis of IrUGT86A1-like Protein from I. rubescens

**Figure 3:**
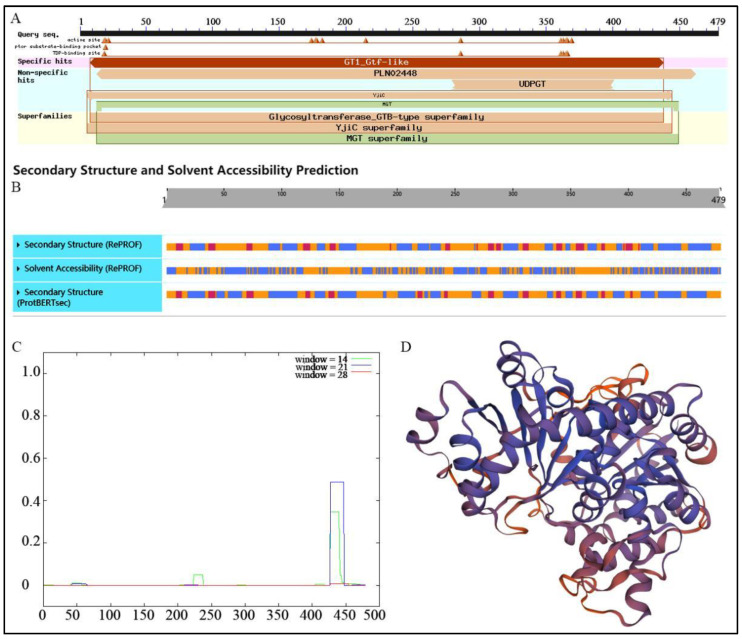
Structural analysis of IrUGT86A1-like protein. Conserved domains (**A**), secondary structure (**B**), COILS analysis (**C**), and 3 D structure (**D**) prediction of IrUGT86A1-like protein.

Conserved domains analysis in NCBI (Figure 3A) demonstrated that the protein had GT1_Gtf-like site-specific and non-specific sites of PLN02448 and UDPGT, which indicted that IrUGT86A1-like belongs to the Glycosyltransferase_GTB-type superfamily. The predicted secondary structure of the IrUGT86A1-like protein according to PredictProtein is shown in Figure 3B, in which 33.82% of the secondary structure is composed of and alpha-helix structure (H, α-helix), 11.27% is strand structure (E, β-sheet), and the remaining 54.91% is categorized as “other structure”. Solvent-accessibility predicted that 38.41% of the protein is exposed, 54.91% is buried, and 6.68% is intermediate. Therefore, these results predicted that IrUGT86A1-like protein is a mixed protein. The COILS prediction indicates that there are seven coiled helix regions when window = 14. Moreover, when window = 21, there are three coiled regions. In the case of window = 28, there is a coil region (Figure 3C). Finally, SWISS-MODEL was used to predict the three-dimensional structure of IrUGT86A1-like protein. As shown in Figure 3D, the UGT85H2 (ID: 2pq6.1) of *Medicago truncatula* was used as the template for system modeling, and the modeling range of IrUGT86A1-like protein was 7~465 aa. The model coverage was 95%, sequence similarity was 37%, and identity coincidence was 76.35%. It is evident in the tertiary structure diagram that the structure composition is consistent with the secondary structure, mainly composed of alpha-helix and loop/coil.

### 3.4. Phylogenetic Analysis of IrUGT86A1-like in I. rubescens

**Figure 4:**
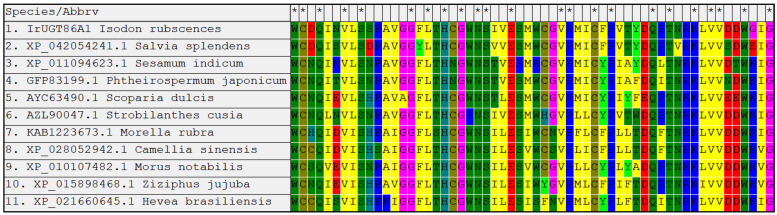
Conserved regions alignment analysis of IrUGT86A1-like protein homologous sequences.

The amino acid sequence of IrUGT86A1-like protein was uploaded and searched against the nr non-redundant protein database with using the online software Protein BLAST from NCBI, and other plant UGT86A1 proteins with high homology to the query amino acid sequence of IrUGT86A1-like in *I. rubescens* were obtained. The amino acid sequences of IrUGT86A1-like from *I. rubescens*, *Salvia splendens*, *Sesamum indicum*, *Phthemospermum japonicum*, *Scoparia dulcis*, *Strobilanthes cusia*, *Morella rubra*, *Camellia sinensis*, *Morus notabilis*, *Ziziphus jujuba*, and *Hevea brasiliensis* were compared with MEGA software. The UGT86A1 protein of different species was found to be highly conserved in the conserved region (Figure 4).

**Figure 5:**
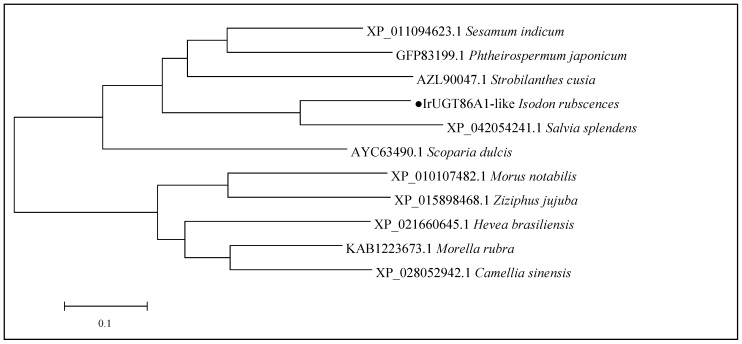
Phylogentic tree construction of IrUGT86A1-like protein. The black dot represents the target IrUGT86A1-like protein.

MEGA7 was further used to construct the phylogenetic tree of the aforementioned 11 UGT86A1 protein sequences, as shown in Figure 5. Phylogenetic analysis revealed that the 11 UGT86A1 protein sequences were generally divided into two branches: woody plants and herbaceous plants. The IrUGT86A1-like protein of *I. rubescens* belongs to the herbaceous branch, grouping with the Labiatae and most closely genetically similar to *Salvia splendens*. Furthermore, the IrUGT86A1-like protein of *I. rubescens* has close genetic relationships with *Strobilanthes cusia*, *Phthemospermum japonicum*, and *Sesamum indicum*. *Scoparia dulcis* is an erect herb or semi-shrub-like herb, also belonging to this branch of herbs. As a transitional stage between herbs and woody plants, *Scoparia dulcis* is closer to woody plants than *I. rubescens*. The evolutionary relationships of the IrUGT86A1-like protein found here are reflective of the evolutionary relationships between herbs and woody plants.

### 3.5. IrUGT86A1-like Expression and Oridonin Content in Different Tissues of I. rubescens

**Figure 6:**
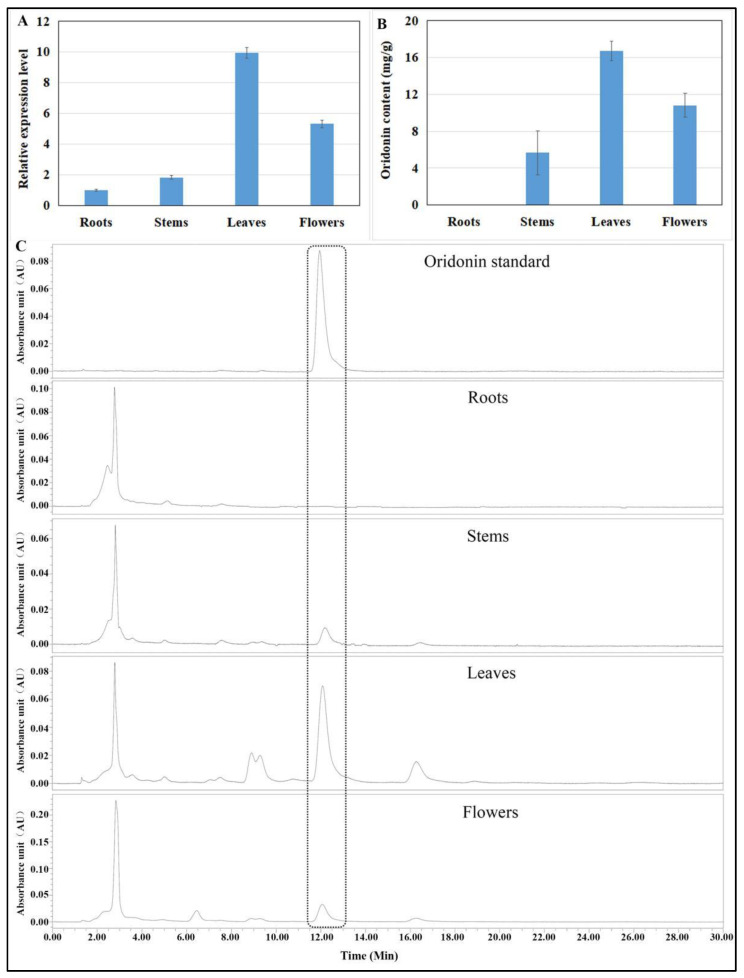
*IrUGT86A1-like* gene expression (**A**), oridonin content (**B**), and HPLC chromatogram (**C**) in different tissues of *I. rubescens*. Results presented by mean value of triplicate ± SE.

The expression of *IrUGT86A1-like* gene in different tissues of *I. rubescens* was detected and measured with RT-qPCR (Figure 6A). Leaves had the highest expression levels of *IrUGT86A1-like* gene, about 9.95-fold than that in roots. Flowers had the second-highest expression levels, about 5.33-fold than that in roots. Stems expressed about 1.84-fold as much as roots.

In addition to the expression profiles of the *IrUGT86A1-like* gene in different tissues of *I. rubescens*, the content of oridonin in these tissues was determined. Leaves contained the greatest oridonin content, followed by flowers, then stems, and roots had the extremely low oridonin content (Figure 6B,C). These results showed that the content of oridonin in different tissues varied with the expression level of the *IrUGT86A1-like* gene in different tissues.

### 3.6. IrUGT86A1-Like Expression under Different Abiotic Treatments of I. rubescens

**Figure 7:**
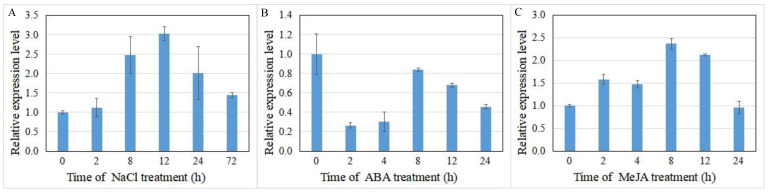
Relative expression analysis of *IrUGT86A1-like* gene at various time periods after abiotic treatments with NaCl (**A**), ABA (**B**), and MeJA (**C**). Results presented by mean value of triplicate ± SE.

Expression of the *UGT* gene has been reported increase in plants subjected to various stress conditions. Herein, the expression of *IrUGT86A1-like* under abiotic stress was further investigated here, and our results demonstrated that *IrUGT86A1-like* expression changed with NaCl, ABA, or MeJA treatments (Figure 7). For NaCl and MeJA treatments, the expression of *IrUGT86A1-like* gene initially increased and then decreased over time. The expression of *IrUGT86A1-like* gene was the greatest after 12 h of NaCl treatment, which was 3.03-fold higher than the baseline expression of the untreated control (Figure 7A). The expression of *IrUGT86A1-like* was the highest after 8 h of MeJA treatment, which was 2.37-fold higher than the untreated control expression level (Figure 7B). In regards to the ABA treatment, the expression of *IrUGT86A1-like* gene was significantly decreased, and the expression was the lowest 2 h after ABA spraying, indicating that *IrUGT86A1-like* gene is sensitive to ABA (Figure 7C).

### 3.7. Prokaryotic Expression Analysis of IrUGT86A1-like Protein in I. rubescens

**Figure 8:**
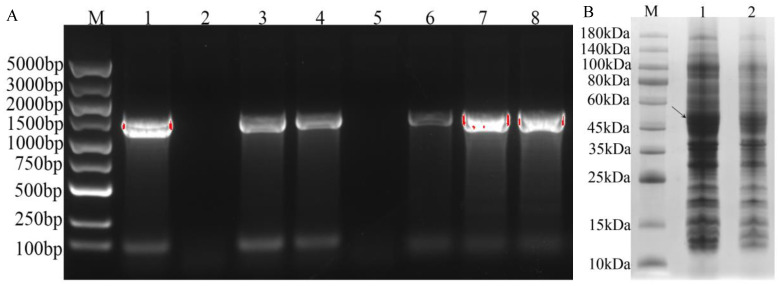
PCR identification and SDS-PAGE analysis of recombinant protein of IrUGT86A1-like. (**A**) PCR identification of recombinant plasmid of IrUGT86A1 (M: DL5000 DNA Maker; 1~8: PCR identification of pEASY-Blunt E1-IrUGT86A colony); (**B**) SDS-PAGE analysis of IrUGT86A recombinant protein (M: Standard molecular weight of protein; 1: pEASY-Blunt E1-IrUGT86A induced by IPTG; 2: pEASY-Blunt E1-IrUGT86A was not induced by IPTG); The arrow indicate the target IrUGT86A1-like protein.

With *E. coli* containing pEASY-Blunt-IrUGT86A1 vector as a template, the target fragment was amplified using IrUGT-YF and IrUGT-YR primers and cloned into the prokaryotic expression vector pEASY^®^-Blunt E1 Expression Vector. PCR gel electrophoresis was performed using primers IrUGT-YF and T7 Terminator. The results demonstrated that the vector which completed the correct direction of connection and transformed into *E. coli* T1 competent culture could amplify a band of 1500 bp by PCR. The bacterial culture of the vector with incorrect connection direction or failure connection could only amplify a band of less than 100 bp or no band, and only the correct band was sent to sequencing verification. The results showed that the prokaryotic expression vector pEASY-Blunt E1-IrUGT86A1 was successfully constructed (Figure 8A). The successful recombinant plasmid pEASY-Blunt E1-IrUGT86A1 was further transformed into BL21 (DE3) prokaryotic expression competent cells.

Furthermore, prokaryotic cells containing the target fragment were induced and expressed. The results of protein electrophoresis are shown in Figure 8B. Consistent with predicted results, the target band of *E. coli* strain induced by IPTG was approximately 53 kDa. Compared with the strain without IPTG induction, the same band was not obviously apparent (Figure 8B, Figure S1). Therefore, the IrUGT86A1-like protein was successfully expressed in *E. coli* BL21, which thus lays an important foundation for further study of the IrUGT86A1-like protein’s modification characteristics.

## 4. Discussion

Secondary metabolites play a major role in the plant response to adversity stresses. In plants, for the most part of secondary metabolites accumulate as glycoconjugates. Glycosylation is one of the most common modifications of secondary metabolites, and is carried out by enzymes called glycosyltransferases. In addition, glycosylation on small molecular metabolites modulates various biological events in plants [10,24]. At present, glycosyltransferase genes with various functions have been cloned, and the corresponding enzyme activation and related functions have been verified [9,10,11,12,13,14,15,16,17,18,19,20]. However, due to the specificity of secondary metabolites in different plants, the functions of glycosyltransferases in different species are species-specific. Therefore, the cloning of glycosyltransferase genes from different species is still a hot topic in the field of secondary metabolic molecules in plants.

In this study, we used transcriptome analysis to screen a *UGT* gene, which had significantly higher expression in the leaves (high oridonin content) than the callus (extremely low oridonin content), and speculated that this gene may be involved in the regulation of oridonin synthesis. Based on the sequence of second-generation transcriptome data, the cDNA of *IrUGT86A1-like* with 1450 bp in length, containing a 1440 bp open reading frame (ORF), which encodes for 479 amino acids was amplified by qPCR. Bioinformatics analysis indicating that the protein encoded by *IrUGT86A1-like* gene is a stable hydrophilic protein with several transmembrane domains. It is speculated that IrUGT86A1-like protein is located in the cytoplasm, which is consistent with previously reported results that glycosylation modification mainly occurs in the endoplasmic reticulum and Golgi apparatus [25]. ExPASy analysis showed that IrUGT86A1-like protein has a molecular weight of 53.03 kD, and the IrUGT86A1-like protein approximately 53 kDa was successfully verified and expressed in *E. coli* BL21, which thus lays an important foundation for further study of the IrUGT86A1-like protein’s modification characteristics.

The blastp comparison results from NCBI showed that the cloned *IrUGT86A1-like* gene had high homology (query coverage greater than 95%) with UDP-glycosyltransferase 86A1-like genes in the database. In addition, conserved domains analysis in NCBI demonstrated that the protein had GT1_Gtf-like site-specific and non-specific sites of PLN02448 and UDPGT, which indicted that IrUGT86A1-like belongs to the Glycosyltransferase_GTB-type Superfamily. Phylogenetic analysis revealed that IrUGT86A1-like protein is most closely genetically similar to *Salvia splendens*. Thus, it is believed that *IrUGT86A1-like* gene belong to the *UGT86* gene subfamily.

Previous studies have shown that glycosyltransferases are involved in a wide range of biological processes essential to plant life, particularly in plant secondary metabolic pathways where they often play an important role in the modification of secondary metabolites to synthesize final compound that the *UGT86C11* acts as a novel plant UDP-glycosyltransferase involved in the products [8,26]. For example, during the synthesis of flavonoids, the glycosylation catalyzed by UDP-glycosyltransferase is a key step at the end of the flavonoid biosynthesis pathway [20,24]. Additionally, it has been shown that glycosylation can improve the stability, diversity, and biological activity of flavonoids [26]. In particular, Payal Srivastavaa et. Al. found synthesis of neoandrographolide and andrograpanin, which belong to the labdane diterpenes [27]. In our study, these results of *IrUGT86A1-like* expression and oridonin content in of *I. rubescens* showed that the content of oridonin in different tissues varied with the expression level of the *IrUGT86A1-like* gene in different tissues. However, although no oridonin content was detected in roots, the *IrUGT86A1-like* gene was expressed in roots. This implies that perhaps *IrUGT86A1-like* gene plays roles in other functions in roots. In addition, congruent relationship of *IrUGT86A1-like* expression and oridonin content are consistent with previous laboratory studies in which the leaves (high oridonin content) had significantly higher expression of *IrUGT86A1-like* than the callus (extremely low oridonin content). In summary, these results suggest that the *IrUGT86A1-like* gene may be involved in the regulation of oridonin synthesis. Furthermore, based on homologous genes that have similar functional properties, the *IrUGT86A1-like* gene, as one of the members of the *UGT86* subfamily, may also be involved in diterpene biosynthesis. Therefore, based on the results of this study, it is speculated that *IrUGT86A1-like* gene may play a regulatory role in the synthesis of oridonin. However, whether *IrUGT86A1-like* gene plays a modification role in oridonin synthesis or not requires further investigation.

In addition, expression of the *UGT* gene has been reported increase in plants subjected to various stress conditions [28,29,30]. Previous studies on *Arabidopsis thaliana* have shown that the expression of *UGT79B2* and *UGT79B3*, could be highly induced by various abiotic stressors such as cold, salt, and drought. Additionally, *UGT75C1*, *UGT78D2*, *UGT79B2*, and *UGT79B3* in *A. thaliana* can make plants more resistant or more sensitive to stress through glycosylation of flavonoids [31]. In addition, previous reports on the *UGT86A1* gene in *A. thaliana UGT86A1* knockout lines and overexpression lines found that the glycosyltransferase gene plays an important role in enhancing the adaptation of plants to abiotic stress by altering the expression of stress-related genes [32]. In our study, it also indicated that *IrUGT86A1-like* gene also played a certain regulatory role in stress regulation. At the same time, abiotic stressors also affect the accumulation of secondary metabolites [33]. Therefore, *IrUGT86A1-like* gene may also be involved in the regulation of oridonin synthesis under the influence of different stresses. Whether the change of *UGT* gene expression under different stressors has a specific mechanistic relationship with the shifts in oridonin abundance needs further verification.

## 5. Conclusions

In this study, the *IrUGT86A1-like* gene of *I. rubescens* has been isolated with RT-PCR and characterized with RNA expression analysis, bioinformatics, and prokaryotic expression. *IrUGT86A1-like* gene encodes for a protein with stable and hydrophilic characteristics, containing a transmembrane domain, and located in the cytoplasm. According to RT-qPCR results, the patterns in expression levels of the *IrUGT86A1-like* gene in different tissues were similar to the distribution of oridonin content in the different tissues of *I. rubescens*, preliminary evidence that *IrUGT86A1-like* gene may be involved in the regulation or modification of oridonin synthesis. Moreover, the expression of *IrUGT86A1-like* gene was found to be altered by abiotic stressors (NaCl, ABA, and MeJA treatments). Expression *IrUGT86A1-like* gene was found to be up-regulated by NaCl and MeJA treatments, and down-regulated by ABA treatment. Finally, prokaryotic expression of IrUGT86A1-like protein successfully expressed the approximately 53 kD IrUGT86A1-like protein, thus contributing an important foundation for further functional research of IrUGT86A1-like protein. Overall, the results of this study provide a theoretical basis for elucidating the potential function of the *IrUGT86A1-like* gene in *I. rubescens*, and enabling future investigation of the function of this gene in the synthesis and modification of secondary metabolites.

## Data Availability

Not applicable.

## Supplementary Materials

The following supporting information can be downloaded at: https://www.mdpi.com/article/10.3390/life12091334/s1, Figure S1: Original Western bolt images of pEASY-Blunt E1-IrUGT86A induced by IPTG (Figure 8B). The marker from top to bottom are 180 kDa, 140 kDa, 100 kDa, 80 kDa, 60 kDa, 45 kDa, 35 kDa, 25 kDa, 15 kDa, 10 kDa.; Table S1: All primers used in this paper.; Table S2: Physicochemical properties and localization analysis of IrUGT86A1-like protein.
