# Supplementary material for: Molecular Cloning and Functional Analysis of IrUGT86A1-like Gene in Medicinal Plant Isodon rubescens (Hemsl.) Hara
Source: Life (Basel). 2022 Aug 28;12(9):1334. doi: 10.3390/life12091334 (PMC9503823; doi:10.3390/life12091334)
Supplement: Supplementary file 1 [file life-12-01334-s001.zip › life-1864674-Supplementary.pdf]

Table S1. All primers used in this paper.

Primer Name	Primer Sequence (5'-3')	Primer Sequence (5'-3')
UGT-F	CCACAGAAAAATGGCGGATG	For *IrUGT86A1-like* gene cloning
UGT-R	CAGGCGAAGCAGCTGCTCTAG	
UDP-qF	CGACTACGACCGTGATGTCC	For RT-qPCR of *IrUGT86A1-like* gene
UDP-qR	TAAACAGTG TCGGCGACCAA	
GAPDH-F	CCCCTCAGACTCCTCCTTGA	For RT-qPCR act as reference gene
GAPDH-R	GGCAAAGTTCTCCCTGCTCT	
UGT-YF	CGACTACGACCGTGATGTCC	For pEASY-Blunt E1-IrUGT86A1 vector construction
UGT-YR	TAAACAGTG TCGGCGACCAA	

Table S2. Physicochemical properties and localization analysis of IrUGT86A1-like protein.

Parameters of IrUGT86A1-like protein	Value
Number of amino acids	479
Molecular weight	53301.72
Theoretical pI	5.33
Total number of negatively charged residues (Asp + Glu)	59
Total number of positively charged residues (Arg + Lys)	41
Formula	$C_{2404}H_{3723}N_{633}O_{708}S_{15}$
Total number of atoms	7483
Instability index	35.16 (stable)
Aliphatic index	94.82
Grand average of hydropathicity (GRAVY)	-0.057
Signal peptide analysis	no
WoLF PSORT predicted subcellular localization	cytoplasm: 8 sites<br>chloroplast: 4 sites<br>nucleus: 2 sites
PredictProtein predicted subcellular localization	cytoplasm

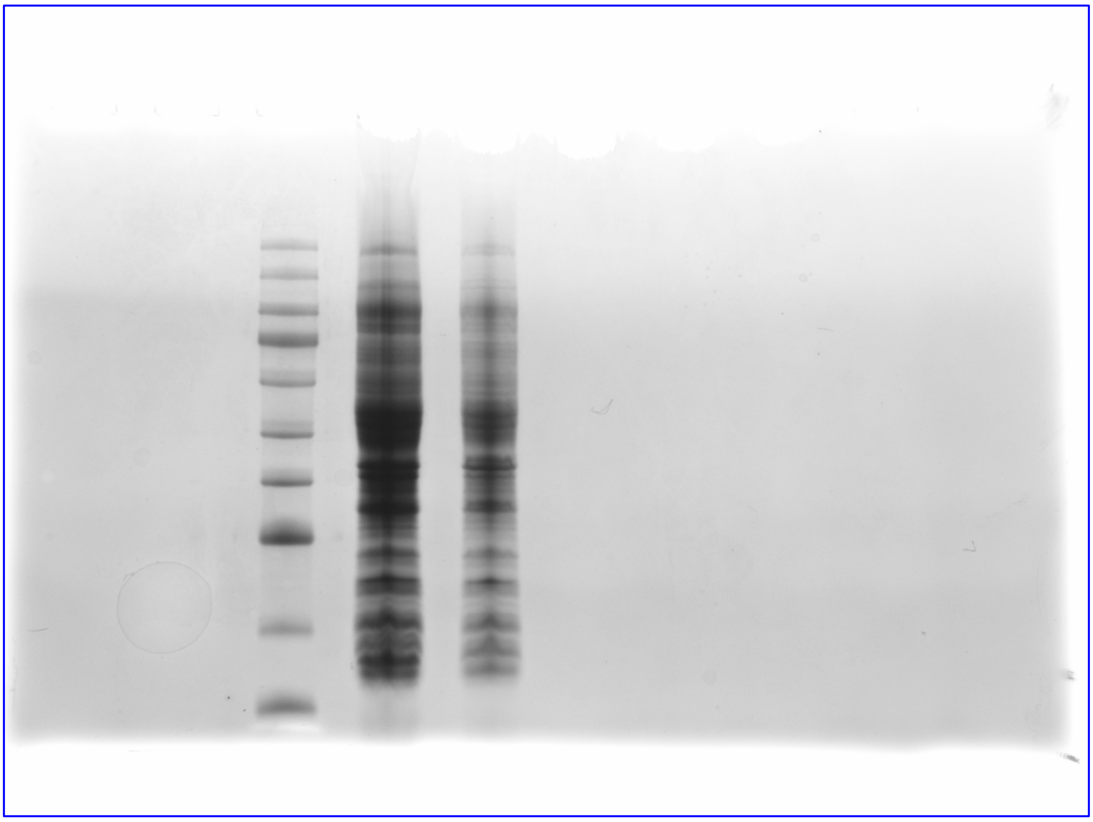

Figure S1. Original Western bolt images of pEASY-Blunt E1-IrUGT86A induced by IPTG (Figure 8B). The marker from top to bottom are 180 kDa, 140 kDa, 100 kDa, 80 kDa, 60 kDa, 45 kDa, 35 kDa, 25 kDa, 15 kDa, 10 kDa.
